# Supplementary material for: Physicochemical and Sensory Assessments in Spain and United States of PGI-Certified Ternera de Navarra vs. Certified Angus Beef
Source: Foods. 2021 Jun 25;10(7):1474. doi: 10.3390/foods10071474 (PMC8303621; doi:10.3390/foods10071474)
Supplement: Supplementary file 1 [file foods-10-01474-s001.zip › foods-1230254-supplementary.pdf]

Table S1. Comparison of minimum specifications between USDA Certified Angus Beef and PGI-Certified *Ternera de Navarra*.

**Program/Brand name**	***Certified Angus Beef***	**PGI Certified *Ternera de Navarra***
Specification number	G-1	N/A
Live Animal Requirement		
Phenotype	AAA	N/A
Genotype	AAA	*Pyreanean* (*Pirenaica*); Blonde d' Aqquitane, Alpine Brown, Charolaise and crossbreds
Other	N/A	Autochthonous of the Navarra region. Mandatory suckling up to 4 mo. of age and regulated feeding practices.
Carcass characteristics		
US Prime	x	N/A
US Choice	x	N/A
Maturity	<30 mo. of age	Male calves: 9 to 13 mo. of age<br>Female calves: 8 to 12 mo. Of age
Marbling Score Requirement	Modest^{00} or higher	N/A
Medium or fine marbling texture	x	N/A
Ribeye area (cm^2)	64.5-103.2/122.6**	N/A
Hot carcass weight (kg)	476.27*	N/A
Fat thickness (cm)	<1.0	N/A
Fat cover class		Between 2 and 3***
Minimum muscling requirement	x	S-E-U-R***
Carcass Class (type)	Steers & Heifers	Veal (Ternera) Eligible for entire male and female calves.
Capillary rupture in ribeye muscle	PF	N/A
Free of dark cutting traits	x	N/A
Max. Hump height <5.08 cm	x	N/A

x=Program requirement. * See specification for specific program requirement.
** G-1 Specification:
Option 1 (default) is 64.5-103.2 cm^2 REA
Option 2 is 64.5-122.6 cm^2 REA.
AAA=USDA Specification for characteristics of cattle eligible for Approved Beef Programs claiming Angus influence. Currently, for phenotypic requirements, cattle must be predominantly (51 percent) black, along with other exclusionary criteria.
PF=practically free of capillary ruptures.
*** Fat cover and conformation scores according to the EU classification norm.
N/A=Not applicable.
Source: USDA Certified Beef Programs
https://www.ams.usda.gov/sites/default/files/media/LPSCertifiedBeefProgramComparison.pdf;
Pliego de condiciones de la IGP "Ternera de Navarra-Nafarroako Aratxea"
http://www.navarra.es/NR/rdonlyres/48025029-AFE9-4F8F-8557-CA727D484637/304236/PCIGPTerneradeNavarra2004.pdf
